# Prevalence of outer retinal tubulation in eyes with choroidal neovascularization

**DOI:** 10.1186/s40942-016-0029-8

**Published:** 2016-03-01

**Authors:** Richard Geraldo Giachetti Filho, Leandro Cabral Zacharias, Thaís Vera Monteiro, Rony Carlos Preti, Sérgio Gianoti Pimentel

**Affiliations:** Hospital das Clínicas da Faculdade de Medicina da Universidade de São Paulo, Av. Dr. Eneas Carvalho de Aguiar, 155-Bloco 8-6º andar, São Paulo, Brazil

**Keywords:** Outer retinal tubulation, Choroidal neovascularization, Spectral-domain optical coherence tomography, Intravitreous injection, Vascular endothelial growth factor, Age-related macular disease

## Abstract

**Background:**

Outer retinal tubulations (ORTs) are branching tubular structures located in the outer nuclear layer of the retina. The goal of this study is to determine the prevalence of ORTs observed in eyes with choroidal neovascularization (CNV) undergoing treatment with anti-angiogenic intravitreous injection (IVI) with anti-VEGF (vascular endothelial growth factor) at the Ophthalmology Department of a tertiary hospital in São Paulo, Brazil.

**Methods:**

This is a descriptive study based on medical charts and Spectral-domain Optical Coherence Tomography (Sd-OCT) scans of 142 patients (158 eyes) treated between 2012 and 2014 with IVI of anti-VEGF for CNV. The patients’ data was analysed according to age, gender, pathology, presence of ORTs, and best corrected visual acuity (BCVA). Patients with and without ORTs were compared according to the last BCVA obtained using Chi square corrected by the Yates factor.

**Results:**

ORTs were found in a total of 40 out of 158 eyes (25.31 %) with CNV; in 33 out of 119 eyes (27.7 %) with neovascular age-related macular disease (AMD); in 5 out of 8 eyes (62.5 %) with neovascular angioid streaks; and in 2 out of 12 eyes (16.67 %) with myopic neovascular membranes. Most patients with ORTs had BCVA worse than 20/200, significantly worse BCVA than patients without ORTs.

**Conclusions:**

Recent studies have considered that the presence of ORTs is indicative of a photoreceptor degeneration process and may represent a final stage of multiple retinal degenerative pathologies. The prevalence of ORTs in eyes with CNV has not been well described, especially when considering the Brazilian population treated in a public health care system. In our study, ORTs were observed in only three different pathologies: neovascular AMD, neovascular angioid streaks and myopic neovascular membranes. The correct recognition of ORTs is fundamental for its differentiation from intraretinal cysts, for the latter is related to the activity of neovascular diseases, and usually guides anti-angiogenic therapy. We conclude that ORTs have a high prevalence in the population studied, and their correct identification presents relevant therapeutic implications.

## Background

The advances in images obtained with spectral-domain optical coherence tomography (SD-OCT) allowed further analysis of retinal and choroidal structures [1].

In 2009, Zweifel et al. [1] first described the “outer retinal tubulation” (ORT), branching tubular structures located in the outer nuclear layer of the retina, which appeared as round or ovoid hyporeflective spaces with a hyperreflective border.

The ORTs were initially observed in eyes with choroidal neovascularization (CNV) secondary to age-related macular degeneration (AMD). More recently, the same alterations were described in other conditions such as retinal and choroidal dystrophies, multifocal choroiditis, central serous choroidopathy, acute zonal occult outer retinopathy and dry AMD with geographic atrophy [1–9].

The importance of ORTs was highlighted by the post hoc analysis of the multicentric comparison of age-related macular degeneration treatments trial (CATT) study [10]. Considering the current definition of ORTs, after a 2-year follow-up of patients with AMD and CNV under anti-angiogenic treatment, this OCT finding was present in 17,4 % of the cases and was related to a worse final visual acuity [10].

The diferentiation of ORTs from intraretinal cysts presents extremely relevant therapeutic implications as the latter are indicative of exsudative activity in diseases such as age-related macular degeneration, diabetic macular edema and vascular occlusions. Specifically for CNV related to AMD, the improvement of visual acuity achieved with monthly intravitreal anti-angiogenic treatment for 24 consecutive months led to the approval of this type of treatment by the FDA [11, 12]. Later, studies such as PRONTO [13] and CATT [10] showed that similar visual acuity could be obtained with fewer intravitreal injections, guided by disease activity parameters such as intraretinal or subretinal fluid detected on OCT.

The aim of this study is to determine the prevalence of ORTs in eyes with CNV undergoing treatment with anti-angiogenic intravitreous injection (IVI) with anti-VEGF (vascular endothelial growth factor) at the Ophthalmology Department of a tertiary hospital in São Paulo, Brazil.

## Methods

This is a descriptive study based on medical charts of patients enrolled at the Ophthalmology Department of the“Hospital das Clinicas”, at the University of São Paulo—São Paulo, Brazil. The SD-OCT scans of the last recorded visit of 142 patients (158 eyes) treated between 2012 and 2014 with IVIs of anti-VEGF for CNV were evaluated. All of these patients were treated with IVIs of bevacizumab, in a pro re nata (PRN) regimen. The images were obtained by the Heidelberg Spectralis (Spectralis Engineering, Heidelberg, Germany) SD-OCT.

**Fig. 1:**
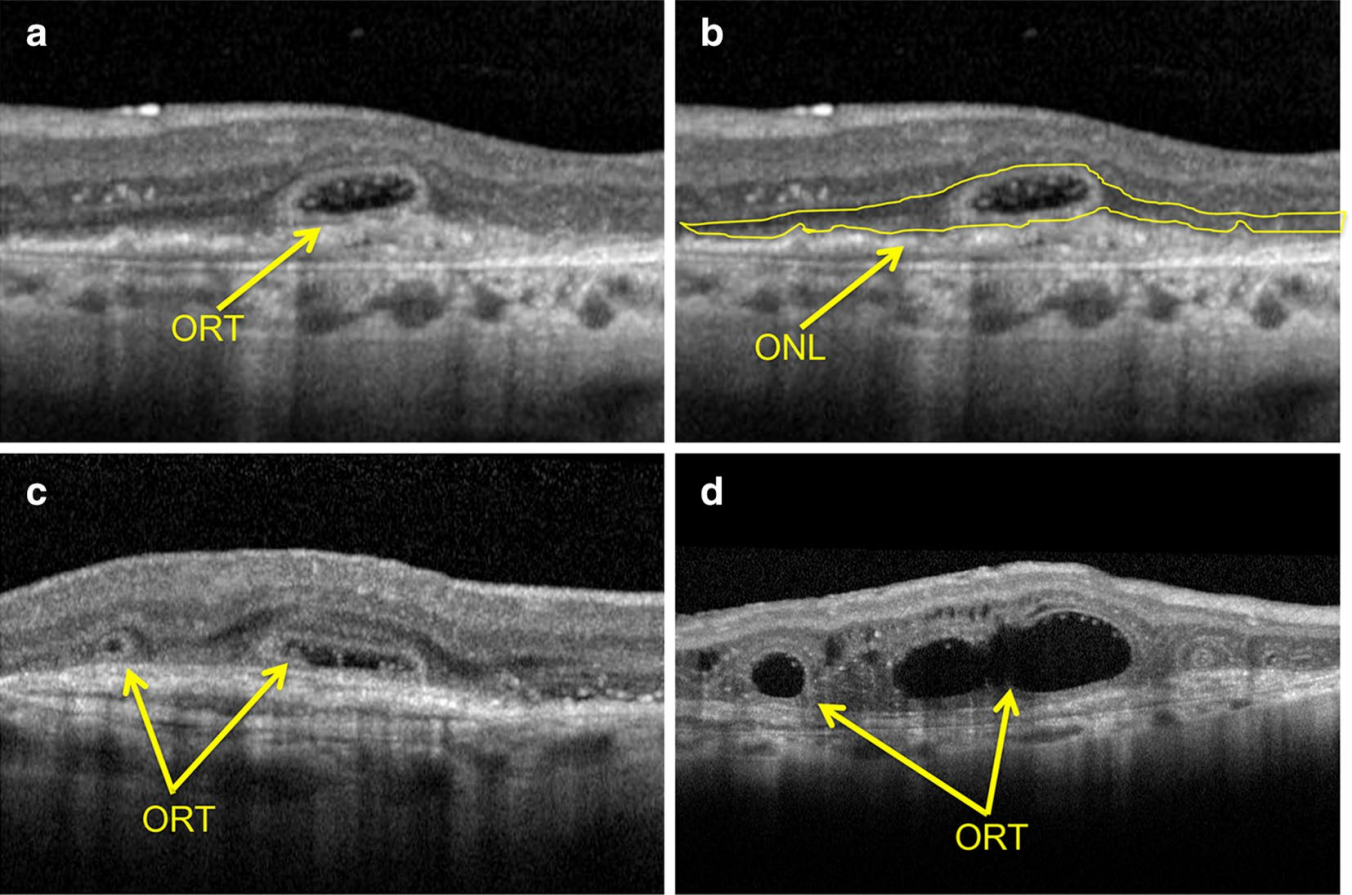
Outer retinal tubulation (ORT) in patients with exsudative AMD treated with IVI anti-VEGF. All images were obtained with the Heidelberg SD-OCT. **a** Annular lesions with hyporeflective lumens and hyperreflective borders at the outer nuclear layer (ONL) **b** ORTs are restricted to the ONL of the retina. **c–d**. Different shapes of ORTs

**Fig. 2:**
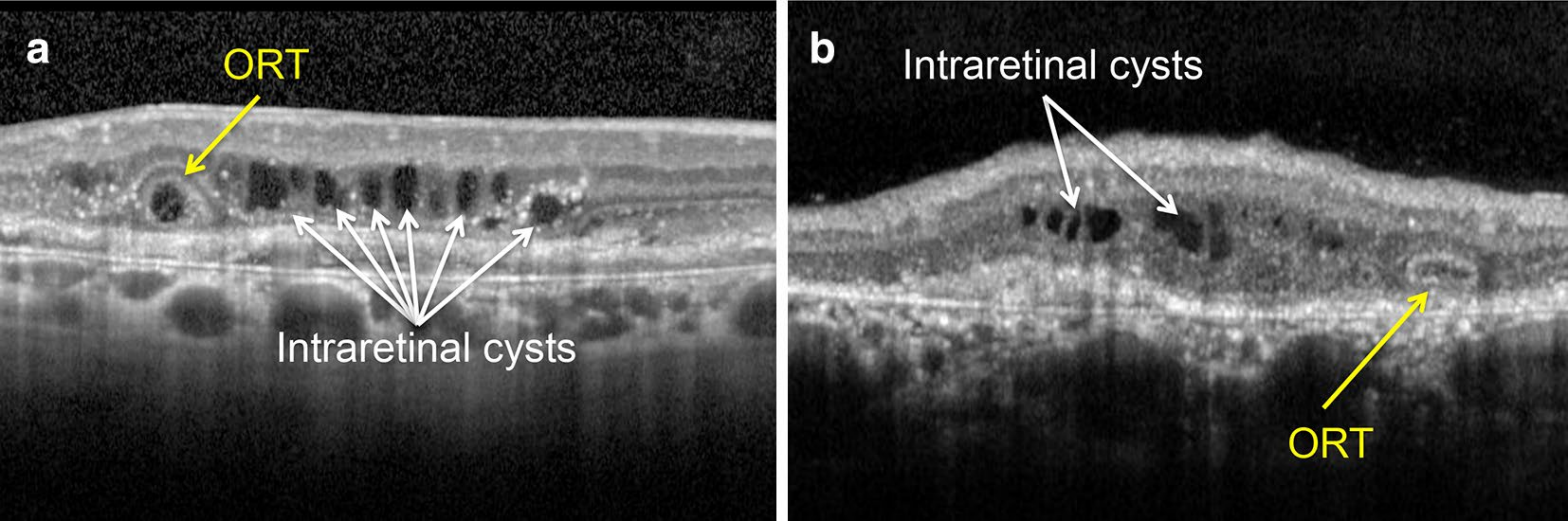
Differentiation between ORT and intraretinal cysts. **a–b**. Next to the circular ORT lesion (with *hyperreflective borders*) there are multiple intraretinal cysts (with *hyporeflective borders*) in the innermost layers of the retina

**Fig. 3:**
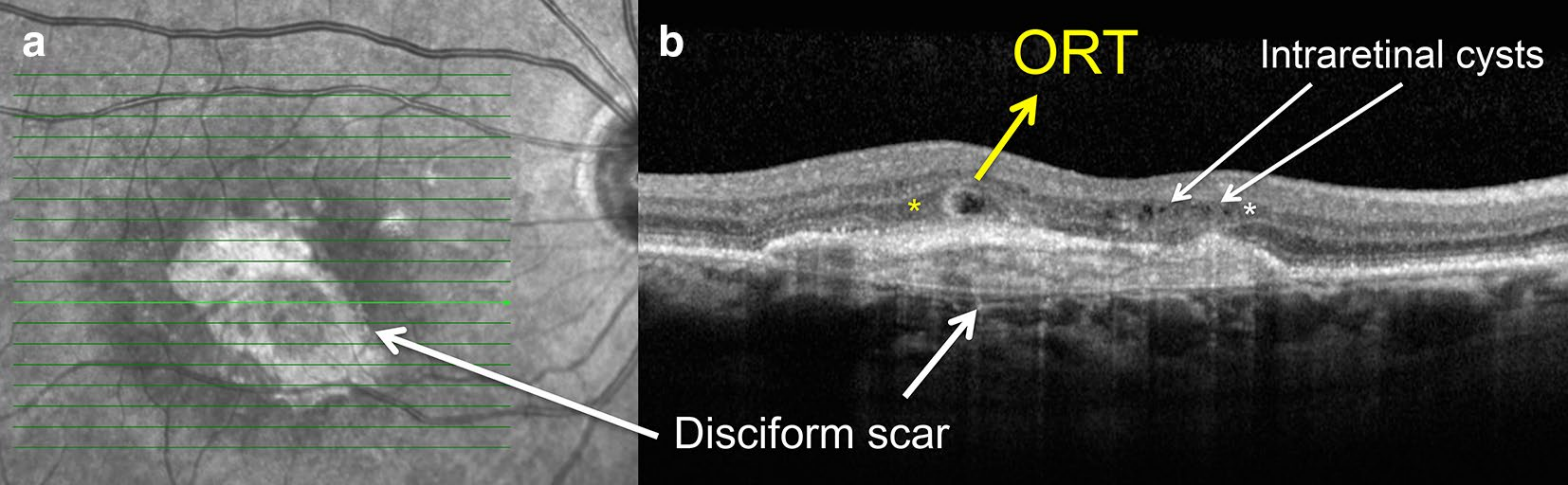
Outer retinal tubulation (ORT) in patient with disciform scar. **a** Infrared image shows a macular lesion. **b** An annular ORT can be observed internally to the disciform scar, in the ONL (*yellow asterisk*); and micro intraretinal cysts are found in the inner nuclear layer (*white asterisk*)

**Fig. 4:**
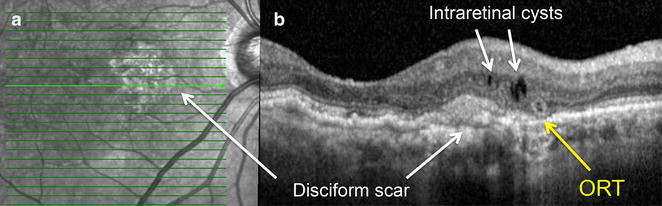
Differentiation between ORT and intraretinal cysts in patient with disciform scar. **a** Infrared image shows a macular lesion. **b** Two intraretinal cysts with hyporeflective borders and an ORT with circular hyperreflective border very close to areas of neovascular fibrosis

ORTs were defined as round or ovoid structures, always with hyperreflective borders, located at the outer nuclear layer (ONL) (Fig. 1), and were differentiated from intraretinal cysts, as the latter are observed at the inner retinal layers and present hyporeflective borders (Figs. 2, 3 and 4).

Image analysis was performed by two retinal specialists. When there was no agreement between examiners, the image was considered inconclusive and therefore not to be an ORT.

The data of patients with CNV was analysed according to age, gender, type of involvement (uni- or bilateral), pathology, presence of ORTs, and best corrected visual acuity (BCVA, early treatment diabetic retinopathy study score) at the final visit (better than 20/40; from 20/40 to 20/200; worse than 20/200). Patients with and without ORTs were compared according to the last BCVA obtained using Chi square corrected by the Yates factor. P < 0.01 was considered as statistically significant. The prevalence of ORTs was estimated by dividing the number of patients with ORTs by the total number of patients examined during the study period.

## Results

A total of 142 patients (158 eyes with CNV) were included in the study. Seventy-nine patients (55.64 %) were women and 59 (44.36 %) were men. The mean age was 58 (24–94 years).

ORTs were present in only three different pathologies with CNV: 33 out of 119 eyes (27.7 %) with neovascular AMD; 5 out of 8 eyes (62.5 %) with neovascular angioid streaks; and 2 out of 12 eyes (16.67 %) with myopic CNV. A total of 40 out of 158 eyes (25.31 %) with CNV presented ORTs.

Other conditions also subjected to IVIs of anti-VEGF for CNV (serpiginous chorioretinopathy, central serous chorioretinopathy, choroidal hemangioma, retinal arterial macroaneurysm, familial drusen, Vogt-Koyanagi-Harada syndrome) did not demonstrate ORTs. (Table 1).

**Table 1:** Demographics and diagnosis of patients with choroidal neovascularization

Disease	n (%) patients	Gender		Age	Eyes	ORTs
		F	M			
Neovascular AMD	105 (73.9 %)	53 (67.3 %)	52 (88.1 %)	76.3 (50–97)	119 (75.4 %)	33 (27.7 %)
Neovascular angioid streaks	7 (4.9 %)	5 (6.3 %)	2 (3.4 %)	60.1 (41–79)	8 (5.0 %)	5 (62.5 %)
VKH syndrome	10 (7 %)	9 (11,4 %)	0	42.5 (25–67)	11 (7.2 %)	0
Myopic CNV	12 (8.4 %)	9 (11.4 %)	0	56.8 (24–94)	12 (7.6 %)	2 (16.67 %)
Polypoidal choroidal vasculopathy	1 (0.7 %)	1 (1.2 %)	0	78	1 (0.6 %)	0
Serpiginous chorioretinopathy	1 (0.7 %)	1 (1.2 %)	0	41	1 (0.6 %)	0
Arterial macroaneurism	1 (0.7 %)	0	1 (1.7 %)	63	1 (0.6 %)	0
Choroidal hemangioma	1 (0.7 %)	0	1 (1.7 %)	51	1 (0.6 %)	0
Central serous chorioretinopathy	3 (2.1 %)	0	3 (5.1 %)	66 (57–77)	3 (1.8 %)	0
Familial drusen	1 (0.7 %)	1 (1.2 %)	0	46	1 (0.6 %)	0
Total	142 (100 %)	79 (100 %)	59 (100 %)	58	158 (100 %)	40

The majority of patients with ORTs had a BCVA worse than 20/200 (57.7 % in neovascular AMD, 60 % in angioid streaks and 50 % in myopic CNV), while those rates were 51.1, 66.7 and 40 %, respectively, for patients without ORTs. (Table 2) Patients with ORTs had a statistically worse BCVA when compared with patients without ORTs (Chi Square equals to 119.8, P < 0.01).

**Table 2:** Comparision of final best corrected visual acuity in patients with or without outer retinal tubulations

	Neovascular AMD		Neovascular angioid streaks		Myopic CNV		Total	
BCVA	Without ORTs	With ORTs	Without ORTs	With ORTs	Without ORTs	With ORTs	Without ORTs	With ORTs
>20/40	6 (7.0 %)	3 (9.0 %)	0	1 (20 %)	3 (30 %)	0	9	4
20/40 a 20/200	44 (51.1 %)	11 (33.3 %)	2 (66.7 %)	1 (20 %)	4 (40 %)	1 (50 %)	50	13
<20/200	36 (41.9 %)	19 (57.7 %)	1 (33.3 %)	3 (60 %)	3 (30 %)	1 (50 %)	40	23
Total	86 (100 %)	33 (100 %)	3 (100 %)	5 (100 %)	10 (100 %)	2 (100 %)	99	40

## Discussion

ORTs were first described by Zweifel et al. [1] in 2009, based on their appearance on SD-OCT scans as round hyporeflective spaces with hyperreflective borders, located in the retinal outer nuclear layer. Those structural changes were only systematically described after the popularization of SD-OCT, as time-domain optical coherence tomography (Stratus OCT, Zeiss, Germany) did not have enough resolution to differentiate them from intraretinal cysts [1].

Initially, ORTs were characterized as interconnecting tubules with degenerated photoreceptors and Müller cells [14]. Recent clinicopathological findings showed that the ORT reflective border represents the mitochondria within degenerated inner segments (IS) translocated internally to the external limiting membrane (ELM), without the presence of outer segments [15, 16].

Recent studies have considered that the presence of ORTs is indicative of a photoreceptor degeneration process and may represent the final stage of multiple advanced retinal diseases, such as: neovascular AMD, polypoidal choroidal vasculopathy (and other causes of CNV); retinal or choroidal dystrophies (gyrate atrophy, choroideremia, cone dystrophy, Bietti crystalline dystrophy, Stargardt disease, pattern dystrophy); central serous choroidopathy and acute zonal occult outer retinopathy [1–9, 17, 18]. The prevalence of ORTs in eyes with CNV has not been well described, especially when considering the Brazilian population treated in a public health care system.

In our study, from the 158 eyes with CNV that received anti-VEGF therapy, the ORTs were only found in three distinct pathologies: neovascular AMD, angioid streaks and myopic CNV, with a high prevalence in the first two conditions (27.7 and 16.67 %, respectively). Such data suggests that those conditions may produce more damage to the photoreceptors that, in an attempt to survive in a hostile environment, tend to form the tubules.

Previous reports have described the high prevalence of ORTs in neovascular AMD (8.2–40 %) [1, 6, 17, 19]. The high proportion of patients with neovascular AMD and ORTs in our study (27.7 %) is similar to the prevalence of ORTs observed in tertiary care centers in Switzerland and France (30 and 38.1 %, respectively) [19, 20], but is higher than the value observed at the CATT study (17.4 %) [10]. The lower prevalence at the CATT trial may be explained by its rigid inclusion criteria that excluded some chronic partially fibrotic cases, differing from a real life scenario such as ours.

The relationship between ORTs and CNV activity and its response to anti-VEGF therapy is still unknown. However, in our cases, eyes with ORTs had significantly worse visual acuity than those without ORTs, possibly because of the association between ORTs and photoreceptor degeneration [10, 17]. Therefore, ORTs may predict disease severity, despite the possibility of having ORTs and BCVA better than 20/40. In some studies, the prevalence of ORT in neovascular AMD and angioid streaks was lower in eyes with better VA (>20/40), suggesting that disease severity may be a trigger to ORT formation [1, 10, 19].

The prevalence of ORTs increases progressively with follow-up despite treatment with anti-VEGF. Darien et al. observed an increase from 2.5 % at baseline to 40 % after a 4-year period of treatment with ranibizumab [19]. A subtle increase was also observed in a post hoc evaluation of the CATT study, when comparing week 52 and week 104. The ORT prevalence increased from 10.1 to 17.4 %, respectively [10]. Interestingly, the regimen of treatments (PRN or monthly treatments) did not alter the prevalence of ORTs.

Recently, new studies involving SD-OCT angiography allow the clinician to visualize CNV noninvasively and may provide a method for identifying and guiding treatment of CNV [21]. It would be interesting if further analysis using this new technology was performed in patients with ORT.

## Conclusions

In conclusion, the prevalence of ORTs in a population with CNV treated with anti-VEGF therapy at a tertiary public health center in Brazil is similar to that found in European studies [19, 20]. Its correct recognition is fundamental for differentiation from intraretinal cysts, frequently an indicative of active neovascular disease, and therefore a guide to anti-angiogenic therapy in PRN regimens. Despite its presence in degenerative processes, the existence of ORTs must not be considered a sign of exsudative disease, but rather a degenerative structural sign. Patients with ORTs usually present a worse BCVA when compared to patients without this finding, but a good visual outcome may be possible even in the presence of this alteration.

## Abbreviations

ORT: outer retinal tubulation
SD-OCT: spectral-domain optical coherence tomography
CNV: choroidal neovascularization
IVI: intravitreous injection
VEGF: vascular endothelial growth factor
BCVA: best corrected visual acuity
AMD: age-related macular disease
IS: inner segments
ELM: external limiting membrane
